# The Cholera in Cincinnati; or a Connected View of the Controversy between the Homœopathists and the Methodist Expositor

**Published:** 1850-05

**Authors:** 


### ARTICLE V.

*The Cholera in Cincinnati: or a connected view of the controversy between the Homœopathists and the Methodist Expositor.—Also a Review of the Report read before the Homœopathic Association.*—By S. A. Latta, M.D. pp. 40. 8 vo.

We cannot go very much into detail in a notice of Dr. Latta's pamphlet; indeed we do not know that it is necessary.—We will however, present our readers with a brief synopsis of it.

Certain persons in the Queen City, *soi disant* doctors, issued a bulletin setting forth that they had, during the late epidemic in that city, Homœopathically treated 1116 cholera patients—of whom 474 were Americans and the remainder principally Germans—and lost but two Americans and thirty-three Germans; and that the remedy they used was strong tincture of Camphor in doses equal to one or two drops every five minutes. Dr. Latta reviewed the report, in the Methodist Expositor, of which he is the editor: setting forth in the first place, that no one but a charlatan would blow his own trumpet in the manner these persons had done; next that according to their own showing, they had abandoned Homœopathy; and lastly, that the report was false. In proof of its falsity he stated that he was cognizant of the fact, that at least nine Americans had died in their hands.

Hereupon the Homœopaths addressed the Doctor a reply which is about as complete a specimen of unlettered rudeness as ever came under our notice; or, to use Dr. Latta's phrase in speaking of it, "it is a confused tissue of insinuations in very bad English." Dr. Latta replied and gave the names and residence of the nine Americans before referred to. This raised a commotion in the Homœopathic camp, and a committee headed by a lawyer and a parson (*par nobile fratrum*) came to the rescue of their beloved leaders. These gentlemen took issue with the Doctor, not only on the question of facts; but, with a confidence in their own extensive acquirements very entertaining to reflect upon, on the subject of the action of remedies. The result of their investigation was to throw a doubt over the statement of Dr. L., so far as three cases were concerned, leaving the other six intact, and of course not helping the case a particle. Pending the investigation of these gentlemen, they were requested to withhold their report for a short time; Dr. Latta promising to furnish them some more statistics. But they very wisely declined his offer, as they doubtless began to fear that the matter would end in demonstrating that their doctors were impostors, and themselves dupes.

In the mean time Dr. Latta furnished the names and residence of nine other American patients who had died in the hands of these doctors, and avers that he has a list of fifty or sixty Germans, which he will add to it when called for.

Mr. Taft (the legal member of the committee) is a queer lawyer, and has peculiar notions of evidence. In making his investigation, he summons the accused doctors, and says, "*Messieurs* you are not guilty, are you?" they indignantly answer no; and on their own testimony he acquits them; for he acknowledges that his evidence is obtained partly from the attending physicians. He also refers to a statute of Ohio, which, Dr. Latta very satisfactorily shows, has no existence. Mr. Taft is thus made to occupy a very ridiculous position, and we feel sorry for him; but he doubtless remembers the maxim of the old Roman law "*ignorantia legum neminem excusat.*" As to the Rev. gentleman who is associated with him, we have only to suggest for his improvement, that text which intimates something in reference to zeal not according to knowledge.

We have no disposition to review the controversy contained in this pamphlet concerning the therapeutic properties of Camphor; as we do not suppose our readers would be thereby edified. Neither is it necessary to follow Dr. Latta's demonstration of the absurdity of these two Homœopaths' assertion that they made probably as many as fifteen thousand professional visits—exclusive of obstetrical and office business—in three months. We therefore close this brief notice with the sincere hope that the inhabitants of Cincinnati may have the good sense to infer that there must be something wrong about a medical theory which requires for its support, unblushing falsehood on the part of its practitioners, and ignorant sophistry and testimony incompatible with all rules of law or logic, on the part of its advocates. M.

